# Predicting mortality in extremely low birth weight infants: Comparison between gestational age, birth weight, Apgar score, CRIB II score, initial and lowest serum albumin levels

**DOI:** 10.1371/journal.pone.0192232

**Published:** 2018-02-13

**Authors:** Jae Hyun Park, Yun Sil Chang, So Yoon Ahn, Se In Sung, Won Soon Park

**Affiliations:** 1 Department of Pediatrics, Dongsan Medical Center, Keimyung University, School of Medicine, Daegu, South Korea; 2 Department of Pediatrics, Samsung Medical Center, Sungkyunkwan University, School of Medicine, Seoul, South Korea; University of North Carolina at Chapel Hill, UNITED STATES

## Abstract

We explored GA, BW, Apgar score, CRIB II score, and serum albumin levels as univariate predictors of mortality in extremely low birth weight infants. Medical records of 564 extremely low birth weight infants were reviewed retrospectively. The infants were grouped as survivors (group I), expired ≤ 7^th^ postnatal day (group II), and expired > 7^th^ postnatal day (group III). The predictive value for mortality of gestational age, birth weight, Apgar scores at 1 and 5 min, clinical risk index for babies II score, and first and lowest serum albumin levels was assessed by calculating the associated area under the curve (AUC) in receiver operating characteristic (ROC) curves. The overall survival and mortality rates of groups I, II, and III were 81.0% (457/564), 7.6% (43/564), and 11.4% (64/564), respectively. Birth weight, Apgar scores at 1 and 5 min, and first serum albumin levels were significantly higher, while the clinical risk index for babies II score was significantly lower in group I when compared to groups II and III. Gestational age and lowest serum albumin level in group I were significantly higher than group III, but not group II. However, gestational age, birth weight, and clinical risk index for babies II score showed gestational age dependent variations regardless of survival or mortality. Apgar score at 5 min (0.756) and lowest serum albumin level (0.771) demonstrated the highest AUC of the ROC curve in predicting mortality in group II and III, respectively. In conclusion, Apgar score at 5 min and lowest serum albumin level were the most effective predictors for mortality in extremely low birth weight infants during ≤ 7^th^ and > 7^th^ postnatal days, respectively.

## Introduction

Despite recent advances in neonatal intensive care medicine, prematurity remains the major cause of mortality in the newborn infants [1]. Among premature infants, mortality risks are the highest and survival is uncertain in the extremely preterm infants with gestational age (GA) < 28 weeks and extremely low birth weight infants (ELBWIs) with birth weight (BW) < 1000 g [2–7]. Therefore, neonatologists taking care of these ELBWIs and their parents often face difficulties in making decisions—during each stage of the neonatal intensive care—such as whether resuscitation, mechanical ventilation, or other invasive treatments should be initiated, as well as when these treatments should be withdrawn. A more precise prediction of the outcome would ease making these difficult decisions. Accordingly, prediction of mortality in ELBWIs before their discharge is of the utmost clinical importance.

In addition to the significant univariate predictors of neonatal mortality such as GA [8], BW [8], Apgar score [8], initial [9, 10], and lowest serum albumin levels [9], a variety of risk adjustment scores such as clinical risk index for babies II (CRIB II) score—which assesses 5 combined variables of sex, GA, BW, body temperature, and base excess—have been found to be good predictors for mortality in preterm infants before 32 weeks of gestation [11]. The relationship of these variables with mortality is in need of further investigation in ELBWIs near the viability limit. We thus conducted this study to explore GA, BW, Apgar score, CRIB II score, and serum albumin levels as univariate predictors of mortality, and to explore possible differences in cause of death in infants that die shortly after birth (≤ 7^th^ postnatal day) versus later (> 7^th^ postnatal day to NICU discharge).

## Materials and methods

### Patients

Data collection was approved by the Institutional Review Board of Samsung Medical Center, and the requirement for informed consent was waived in this retrospective chart review (IRB No. SMC 2016-03-122). Medical records were reviewed retrospectively for 564 ELBWIs with BW < 1,000 g, who were admitted to the neonatal intensive care unit (NICU) of Samsung Medical Center after birth from January 1, 2001 to December 31, 2011. The infants were stratified into gestation subgroups of 23–24, 25–26, and ≥ 27 weeks gestation. These infants were then divided into three subgroups based on their outcomes: survivors (group I), those who expired ≤ 7^th^ postnatal day (group II), and those who expired > 7^th^ postnatal day (group III).

Variables including GA, BW, sex, and Apgar scores at 1 and 5 min were analyzed. GA was determined by maternal last menstrual period and modified Ballard test. First serum albumin level measured within first 3 postnatal days and lowest serum albumin level measured during admission were obtained from medical records. CRIB II score was calculated by five variables of sex, GA, BW, base excess, and body temperature measured upon admission to NICU. Base excess was obtained from the first blood gas analysis by retrospective chart review.

Cause of death—defined as the disorder directly and immediately causing death of the infant—was ascertained from the death certificate, and the causes of death were adjudicated with the information obtained from the medical records. These listed disorders were not categorized, and differences in the proportion of infants in group II and group III who died from each cause were tested with a chi-square test. There was no death directly and immediately caused by major congenital anomalies in these infants. Pulmonary hypoplasia was defined as clinical findings associated with oligohydramnios from premature rupture of membrane plus aggressive ventilator support, but was not histologically confirmed. Air leak syndrome was defined as radiologic findings of extra-pulmonary air requiring chest tube insertion and drainage. Pulmonary hemorrhage was defined as presenting with bloody fluid from the endotracheal tube plus radiologic suggestion of pulmonary hemorrhage. Acute renal failure was defined as urine output of < 0.5 mL/kg/day for 24 hours combined with a serum creatinine level of 2.0 mg/dL. Sepsis was defined as clinical symptoms or signs suggestive of sepsis including hypotension plus positive blood culture. Bronchopulmonary dysplasia (BPD) was defined as the need for supplemental oxygen or positive pressure to maintain oxygen saturation greater than 90% at 28 postnatal days [12]. Intraventricular hemorrhage (IVH) was defined as grade III or higher IVH or the ensuing post-hemorrhagic hydrocephalus [13], and necrotizing enterocolitis (NEC) was defined as modified Bell’s stage IIb [14].

### Statistical analysis

Continuous variables are expressed as mean ± standard deviation, and median (interquatile range, IQR). Categorical variables are expressed as number and percentage. Comparisons between categorical variables were performed using Chi-square test, and comparisons between continuous variables were evaluated using one-way analysis of variance (ANOVA). A p-value of < 0.05 was considered statistically significant. The software package SPSS version 20 (SPSS Inc., Chicago, IL, USA) was used.

Analysis of the area under the curve (AUC) by calculating the receiver operating characteristics (ROC) curve was performed to assess the discrimination ability to predict the mortality of infants according to timing of death. It was compared with GA, BW, Apgar scores, CRIB II score, and first and lowest serum albumin levels with the selection of the most suitable cut-off point of each variables with the best sensitivity, specificity, positive predictive value, and negative predictive value using the MedCalc. Ver. 15.8 statistical software package (MedCalc Software Mariakerke, Belgium). An AUC score of 0.5 does not reflect any discrimination. The AUC results were considered excellent for AUC values between 0.9–1, good for AUC values between 0.8–0.9, fair for AUC values between 0.7–0.8, poor for AUC values between 0.6–0.7, and failed for AUC values between 0.5–0.6.

## Results

### Mortality rate according to GA

**Fig 1:**
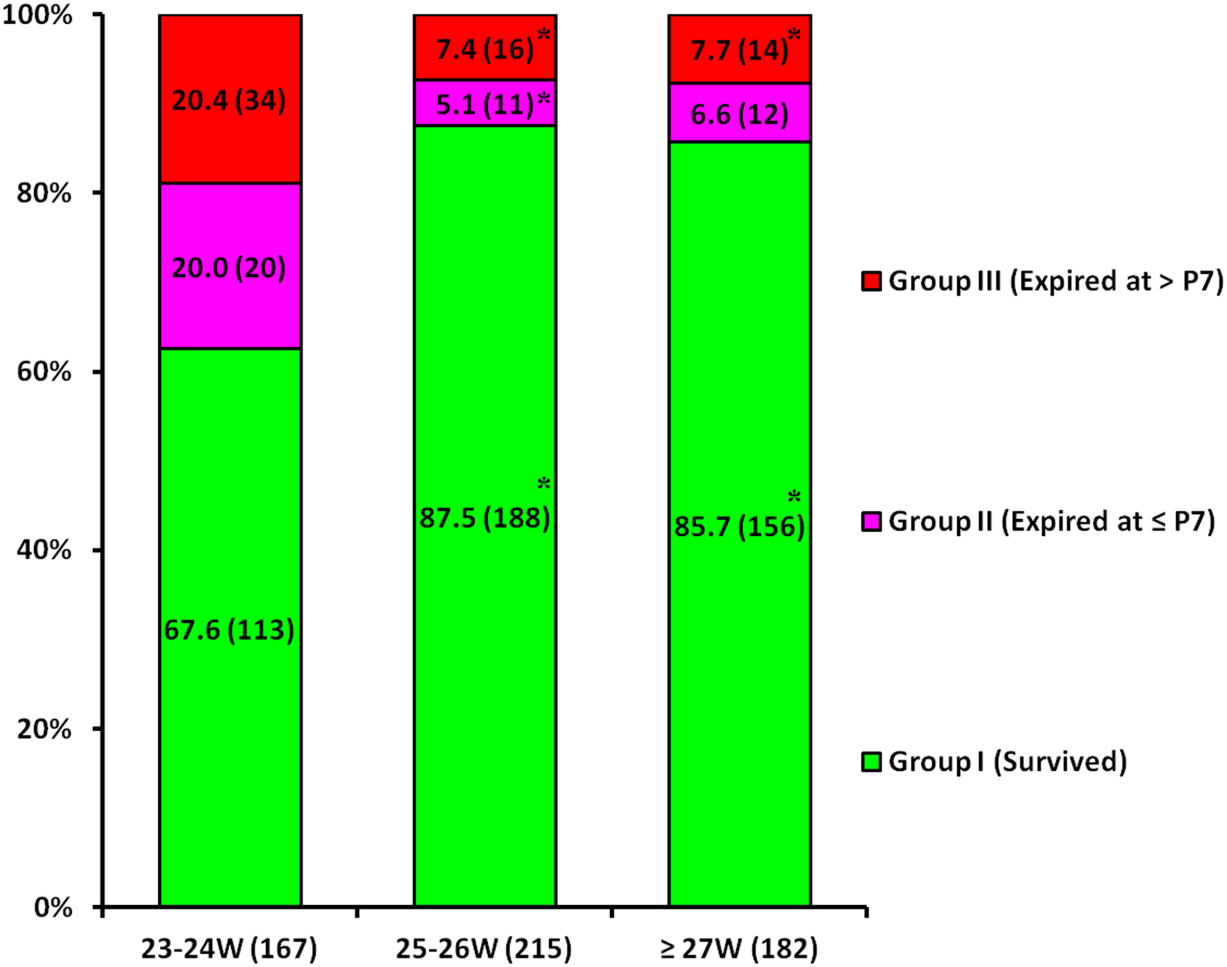
Comparison of survival and mortality according to gestational age. * *p* < 0.05 compared to 23–24 W.

Fig 1 illustrates the survival (group I) and mortality of group II and group III stratified according to GA. Group I was significantly lower, and group II and III were significantly higher in the infants with 23–24 weeks of gestation compared with the infants with 25–26 weeks and ≥ 27 weeks of gestation.

### Cause-specific mortality rate according to GA

Table 1 demonstrates cause-specific mortality rate according to timing of death in the infants stratified according to GA. While the overall mortality due to sepsis was not significantly different between groups II and III, the overall mortality due to pulmonary hypoplasia or air-leak syndrome in the infants with 23–24 weeks of gestation and due to pulmonary hypoplasia in the infants with 25–26 weeks of gestation was significantly higher. The overall mortality due to BPD and NEC was significantly lower in group II compared with group III. According to the definition of BPD [12], we described that there was no occurrence of BPD in group II. The average (IQR) length of NICU stay in terms of timing of death was 96.9 (93.8–99.1) days in group I (survived), 2.8 (2.2–3.4) days in group II (expired ≤ 7 days), and 81.7 (56.8–106.7) days in group III (expired > 7 days).

**Table 1:** Cause specific mortality rate in terms of timing of death (group II: Expired ≤ 7 days, group III: Expired > 7 days) in infants according to gestational age.

Cause of Death, N (%)	23-24Wk (N = 167)			25-26Wk (N = 215)			≥27Wk (N = 182)			Total (N = 564)		
	Group II	Group III	*p* value	Group II	Group III	*p* value	Group II	Group III	*p* value	Group II	Group III	*p* value
	(N = 20)	(N = 34)		(N = 11)	(N = 16)		(N = 12)	(N = 14)		(N = 43)	(N = 64)	
**Sepsis**	5 (25.0%)	9 (26.5%)	0.905	0	2 (12.5%)	0.223	4 (33.3%)	4 (28.6%)	0.793	9 (20.9%)	15 (23.4%)	0.760
**Bronchopulmonary dyspliasia**	0	11 (32.4%)	0.004	0	1 (6.2%)	0.398	0	4 (28.6%)	0.044	0	16 (25.0%)	0.000
**Air leak syndrome**	4 (20.0%)	0	0.007	2 (18.2%)	2 (12.5%)	0.683	0	1 (7.1%)	0.345	6 (14.0%)	3 (4.7%)	0.090
**Pulmonary hypoplasia**	4 (20.0%)	1 (2.9%)	0.037	1 (9.1%)	0	0.219	3 (25.0%)	1 (7.1%)	0.208	8 (18.6%)	2 (3.1%)	0.007
**Pulmonary hemorrhage**	1 (5.0%)	4 (11.8%)	0.408	4 (36.4%)	0	0.009	2 (16.7%)	0	0.112	7 (16.3%)	4 (6.2%)	0.094
**Intraventricular hemorrhage**	4 (20.0%)	4 (11.8%)	0.411	2 (18.2%)	3 (18.8%)	0.970	0	1 (7.1%)	0.345	6 (14.0%)	8 (12.5%)	0.827
**Necrotizing enterocolitis**	0	3 (8.8%)	0.172	1 (9.1%)	5 (31.2%)	0.174	0	1 (7.1%)	0.345	1 (2.3%)	9 (14.1%)	0.041
**Acute renal failure**	1 (5.0%)	0	0.188	0	2 (12.5%)	0.223	1 (8.3%)	1 (7.1%)	0.910	2 (4.7%)	3 (4.7%)	0.993

Data are analyzed using the Chi-square test, and presented as a number with the percentage in parenthesis.

### GA, BW, Apgar score, CRIB II score, and serum albumin levels

Table 2 shows GA, BW, Apgar scores at 1 and 5 min, CRIB II score, and first and lowest serum albumin levels in 3 subgroups according to GA. The overall GA and lowest serum albumin level in group III and BW, Apgar scores at 1 and 5 min, CRIB II score, and first serum albumin level in both groups II and III were significantly lower compared with group I. While significant variations according to GA were observed in all parameters in group I, these GA dependent variations in group II and III were not observed in the parameters of Apgar scores at 1 and 5 min and first and lowest serum albumin levels. The average (IQR) age of lowest serum albumin was 16.2 (14.7–17.7) days in group I, 18.4 (11.2–25.7) days in group III. The average (IQR) time interval from lowest albumin to death in group III was 65.4 (40.9–89.8) days.

**Table 2:** Comparison of mean gestational age, birth weight, Apgar score, CRIB-II score, first and lowest serum albumin levels in 3 subgroups according to gestational age.

		Group I (Survived)	Group II (Expired at ≤ P7)	Group III (Expired at > P7)
**Gestational age (Week)**	**TOTAL**	26.4±2.2	25.9±2.4	25.6±2.1*
**Birth weight (g)**	**23-24W**	658±100	594±103*	615±103*
	**25-26W**	820±114^#^	758±152^#^	670±184*
	**≥ 27W**	847±119^#^	822±131^#^	769±200^#^
	**TOTAL**	789±135	699±159*	663±161*
**Apgar score, 1min**	**23-24W**	4.0±1.5	2.9±1.4*	3.3±1.6*
	**25-26W**	4.6±1.7^#^	2.8±2.2*	4.1±1.8
	**≥ 27W**	4.8±1.7^#^	3.7±1.4*	4.2±1.2
	**TOTAL**	4.5±1.7	3.1±1.7*	3.7±1.6*
**Apgar score, 5min**	**23-24W**	6.8±1.5	5.6±2.0*	6.0±1.7*
	**25-26W**	7.1±1.5	4.1±3.1*	6.6±1.7
	**≥ 27W**	7.4±1.1^#^	6.3±1.3*	7.1±1.0
	**TOTAL**	7.1±1.4	5.4±2.3*	6.4±1.6*
**CRIB-II score**	**23-24W**	15.4±1.7	16.3±1.8*	15.9±1.6
	**25-26W**	12.2±1.6^#^	14.3±2.2*	13.3±1.5* ^#^
	**≥ 27W**	9.7±1.5^#^^$^	10.7±1.9 ^#^^$^	10.5±1.9 ^#^^$^
	**TOTAL**	12.2±2.7	14.1±3.1*	14.2±2.7*
**First albumin (g/dL)**	**23-24W**	2.4±0.3	2.4±0.3	2.4±0.4
	**25-26W**	2.6±0.3^#^	2.4±0.3	2.3±0.4*
	**≥ 27W**	2.8±0.4^#^^$^	2.6±0.7	2.7±0.5
	**TOTAL**	2.6±0.4	2.4±0.5*	2.4±0.4*
**Lowest albumin (g/dL)**	**23-24W**	2.1±0.3	2.2±0.5	1.9±0.4*
	**25-26W**	2.3±0.3^#^	2.4±0.3	2.0±0.5*
	**≥ 27W**	2.4±0.4^#^^$^	2.4±0.8	1.8±0.6*
	**TOTAL**	2.3±0.4	2.3±0.6	1.9±0.5*

Data are analyzed using one-way analysis of variance, and presented as the mean ± standard deviation.

* *p* < 0.05 compared to Group I,

^#^ *p* < 0.05 compared to 23–24 W within same subgroup,

^$^ *p* < 0.05 compared to 25-26W within same subgroup

### ROC curves of variables for predicting mortality

Tables 3 and 4 demonstrate ROC curves of variables for predicting mortality between groups I and II (Table 3), and group I and III (Table 4), respectively. The best mortality predictor in group II was Apgar score at 5 min, which the cut-off value was 6 (AUC 0.756, Sensitivity 62.8%, Specificity 78.4%). The best mortality predictor in group III was lowest serum albumin levels, which the cut-off value was 1.9 mg/dL (AUC 0.771, Sensitivity 73.0%, Specificity 70.0%).

**Table 3:** Receiver operating characteristic curves of gestational age, birth weight, Apgar score at 1- and 5-min, CRIB-II score, first and lowest serum albumin levels for predicting mortality between Group I (survived infants) and group II (infants who expired ≤ 7 days).

Variables	Area under curve+SEM (95% CI)	Cutoff value	Sensitivity-% (95% CI)	Specificity-% (95% CI)	(+)PV	(-)PV
**Gestational age (week)**	0.579 (0.539–0.620)	≤24	46.5 (31.2–62.3)	71.8 (67.7–15.6)	12.0	94.2
**Birth weight (g)**	0.637 (0.596–0.677)	≤700	53.5 (37.7–68.8)	67.2 (63.0–71.2)	11.9	94.6
**Apgar score 1min**	0.707 (0.667–0.744)	≤4	83.7 (69.3–93.2)	47.7 (43.3–52.1)	11.7	97.2
**Apgar score 5min**	0.756 (0.718–0.791)	≤6	62.8 (46.7–77.0)	78.4 (74.6–81.8)	19.4	96.2
**CRIB-II score**	0.667 (0.626–0.707)	>14	53.9 (37.2–69.9)	76.5 (72.6–80.2)	15.0	95.6
**First albumin**	0.624 (0.582–0.665)	≤2.4	51.5 (33.5–69.2)	63.0 (58.7–67.2)	8.1	95.3
**Lowest albumin**	0.531 (0.489–0.573)	>1.9	81.8 (64.5–93.0)	20.2 (16.9–23.9)	6.1	94.6

(+)PV: Positive predictive value;

(-)PV: Negative predictive value; Plus-minus values are means+SEM; Positive and negative predictive values refer to the observed neonatal mortality rate (19.0%).

**Table 4:** Receiver operating characteristic curves of gestational age, birth weight, Apgar score at 1- and 5-min, CRIB-II score, first and lowest serum albumin levels for predicting mortality between group I (survived infants) and group III (infants who expired > 7 days).

Variables	Area under curve+SEM (95% CI)	Cutoff value	Sensitivity-% (95% CI)	Specificity-% (95% CI)	(+)PV	(-)PV
**Gestational age (week)**	0.635 0.592–0.676	≤24	52.3 (39.5–64.9)	75.2 (71.0–79.1)	23.1	91.7
**Birth weight (g)**	0.725 0.684–0.763	≤700	64.6 (51.8–76.1)	71.7 (67.3–75.8)	24.6	93.4
**Apgar score 1min**	0.643 0.600–0.684	≤4	71.9 (59.2–82.4)	50.4 (45.7–55.1)	17.0	92.7
**Apgar score 5min**	0.657 0.614–0.698	≤6	40.6 (28.5–53.6)	81.0 (77.1–84.6)	23.2	90.6
**CRIB-II score**	0.708 0.666–0.747	>14	50.0 (37.0–63.0)	820.2 (76.2–83.8)	26.1	92.0
**First albumin**	0.619 0.576–0.661	≤2.4	64.0 (40.9–66.6)	65.4 (60.8–69.7)	17.7	91.1
**Lowest albumin**	0.771 0.733–0.807	≤1.9	50.8 (37.9–63.6)	84.0 (80.3–87.2)	30.5	92.5

(+)PV: Positive predictive value;

(-)PV: Negative predictive value; Plus-minus values are means+SEM; Positive and negative predictive values refer to the observed neonatal mortality rate (19.0%).

## Discussion

Prediction of mortality in ELBWIs at lowest limits of viability, where clinical judgement is most difficult, would be very helpful for clinical decision making [8]. In the present study, we observed the mortality in the infants with 23–24 weeks of gestation was significantly higher than the infants with 25–26 and ≥27 weeks of gestation. However, in our recent study about the trends in mortality in extremely preterm infants with 23–26 weeks of gestation, we observed that mortality rate only in the most immature infants with 23–24 weeks, but not with 25–26 weeks of gestation was significantly improved [2]. These findings suggest that mortality even among the most immature infants at the verge of viability could be significantly improved by active neonatal intensive care [2, 15, 16]. In addition, we also observed that BPD incidence at infants with 25–26 weeks of gestation was significantly decreased due to early extubation, prolonged use of less invasive continuous positive airway pressure, and reduced supplemental oxygen [16]. In the present study, we thus stratified the infants into 23–24, 25–26, and ≥27 weeks of gestation to explore GA, BW, Apgar score, CRIB II score, and serum albumin levels as univariate predictors of mortality.

The cause of death was variable according to the timing of death observed in the present and other studies [2–7] showing pulmonary causes including air-leak syndrome and pulmonary hypoplasia, and BPD and NEC as the leading cause of death during ≤ 7^th^ and > 7^th^ postnatal days, respectively. The variability of the cause of death according to the timing of death suggests that the parameters predicting mortality might also consequently vary according to the timing of death. In the present study, we thus tested the validity of each variable for predicting mortality during ≤ 7^th^ and > 7^th^ postnatal day separately.

As immaturity and low BW are the well-known leading causes of death in extremely preterm infants [6], GA and BW might be the most effective parameters for predicting mortality in ELBWIs [8]. In the present study, besides significantly higher mortality rate in the infants at 23–24 weeks of gestation than the infants at 25–26 and ≥27 weeks of gestation, the overall GA and BW in survivors was significantly higher compared with the infants expired during both the ≤ 7^th^ and > 7^th^ postnatal days. However, GA and BW in the infants at 23–24 weeks of gestation were significantly lower compared with the infants at both 25–26 and ≥27 weeks of gestation regardless of survival and mortality. The AUC of the ROC curve of GA in the infants who expired during ≤ 7^th^ and > 7^th^ postnatal day were 0.579 and 0.635 respectively, indicating poor predictability of mortality. Overall, these findings suggest that GA and BW weight might not be the most effective universal parameter for predicting mortality in ELBWIs during both ≤ 7^th^ and > 7^th^ postnatal day.

To assess the validity of various parameters for predicting mortality in ELBWIs, it is important to identify their timing and cause-specific death. Historically, most ELBWIs died within first several days of life [3–5] primarily due to immaturity and pulmonary causes [6]. However, Patel et al. [7] reported a significant decline in overall mortality of extremely preterm infants mostly due to decreased deaths related to immaturity and pulmonary causes. We also reported significantly improved mortality in the infants at 23–24 weeks of gestation primarily attributable to reduced deaths due to air-leak syndrome and sepsis during the second through seventh postnatal days [2]. These findings suggest that mortality especially during ≤7^th^ postnatal day even in ELBWIs near the limit of viability is not static and could be significantly improved by providing better neonatal intensive care treatments. Previously, we observed the Apgar score at 5 min was significantly associated with improved mortality during the second through seventh postnatal days in multivariate analysis [2]. In the present study, the Apgar score at 5 min in survivors was significantly higher than the expired infants regardless of GA. Moreover, the AUC of the ROC curve of the Apgar score at 5 min with cutoff value of ≤ 6 in the infants expired during ≤ 7^th^ postnatal day was highest of 0.756; this did not follow for the infants expired during > 7^th^ postnatal day, where the AUC of the ROC curve was 0.657. Taken together, these findings suggest that improved delivery room management evidenced by a better Apgar score at 5 min is primarily responsible for better neonatal intensive care, and the Apgar score at 5 min thus could be the best predictor of mortality during ≤ 7^th^ postnatal day due to pulmonary causes such as air leak syndrome, pulmonary hypoplasia, and pulmonary hemorrhage in ELBWIs.

Hypoalbuminemia has been known to be a strong independent predictor of poor outcome in adults [17], and the lowest serum albumin level showed significant inverse correlation in ELBWIs [9]. As albumin concentration rises with increasing GA [18, 19], even in survivors of ELBWIs observed in the present study, setting the precise reference ranges for pathological hypoalbuminemia playing a prognostic role has proved difficult to determine in ELBWIs. In the present study, the lowest serum albumin level was observed in the ELBWIs who expired during > 7^th^ postnatal day, but not in those who expired during ≤ 7^th^ postnatal day, was significantly lower compared with the survivors. Moreover, the AUC of the ROC curve of the lowest serum albumin level with cutoff value of ≤ 1.9 in the infants expired during > 7^th^ postnatal day was highest of 0.771; however, this did not follow for infants expired during ≤ 7^th^ postnatal day, where the AUC of the ROC curve was 0.531. Overall, these findings suggest that lowest serum albumin level could be the best predictor of mortality during > 7^th^ postnatal day in ELBWIs. The extent of hypoalbuminemia might be just a marker of capillary leakage severity induced by underlying disorders such as IVH, NEC, or BPD rather than a causative agent for mortality [9]. Therefore, further studies will be necessary to determine whether increasing serum albumin levels by injecting albumins might be beneficial and thus improve the outcome in ELBWIs [17].

CRIB II score, calculated by assessing 5 combined variables of gender, GA, BW, admission body temperature, and first base excess has been known to a better mortality predictor than GA or BW alone in preterm infants before 32 weeks gestation [11, 20]. However, in the present study, the multivariate CRIB II score failed to better predict mortality than its univariate parameters such as GA and BW in ELBWIs during both ≤ 7^th^ and > 7^th^ postnatal day in ELBWIs. These findings suggest the existing mortality prediction models including CRIB II score do not adequately reflect recent improvements in neonatal intensive care and ensuing mortality. Accordingly, further studies will be necessary to develop new multivariate models to better predict mortality in ELBWIs [8, 21, 22].

This study had several limitations including its retrospective design. Moreover, although we reviewed all the medical records to confirm the primary cause of death on the death certificate, sometimes it was difficult to differentiate a single primary cause of death in a complex situation where multiple causes interact. Therefore, the listed cause of death in some cases might have been subjective. Another limitation was that our data were obtained from a single institution, so results obtained in the present study might not be generalizable to other institutions. Nonetheless, a relatively large sample size of 564 ELBWIs born and admitted to a single center with similar baseline characteristics and treatment policies by the same neonatologists might redeem this limitation. However, the small numbers of infants within the subgroups were limited to establish which variables were associated with mortality. And, the factors revealed as significant in a univariate analysis for predictors of survival executed with 564 ELBWIs in a single center of the present study cannot be generally used to attempt to predict survival of any individual infant. To verify this, further larger population-based study would be needed.

## Conclusions

The cause of death was variable according to the timing of death, and was due to pulmonary causes during ≤ 7th postnatal day and due to BPD and NEC during > 7^th^ postnatal day in ELBWIs. The Apgar score at 5 min and the lowest serum albumin level were the best predictors of mortality during ≤ 7^th^ and > 7^th^ postnatal days, respectively.
